# Evaluation of ultrahigh-performance liquid chromatography columns for the analysis of unmodified and antisense oligonucleotides

**DOI:** 10.1007/s00216-014-7959-5

**Published:** 2014-06-18

**Authors:** Sylwia Studzińska, Bogusław Buszewski

**Affiliations:** Faculty of Chemistry, Nicolaus Copernicus University, 7 Gagarin Str., 87-100 Toruń, Poland

**Keywords:** Oligonucleotides, Ultrahigh-performance liquid chromatography, Chromatographic column, Ion-pair chromatography, Separation, Human serum

## Abstract

Ultrahigh-performance liquid chromatography has been used for the separation and analysis of unmodified and modified antisense oligonucleotides. For this reason, we tested various columns of low particle sizes in our analysis of unmodified and phosphorothioate oligonucleotides. The influence of both the type and concentration of ion-pair reagent on the retention of the studied biomolecules was tested. The developed methods were used for separation of unmodified oligonucleotides and to determine antisense oligonucleotides in human serum samples. The results proved that octadecyl and phenyl columns are the most selective in the resolution of oligonucleotides which differ in the position of single nucleotides in the sequence. The phenyl column was selected and applied for the analysis of phosphorothioate oligonucleotides in serum samples. The calibration plots showed good linearity within the test concentration ranges. The intra-day CV of the calibration curve slopes was in the range of 1.6 to 4.2 %. The limits of detection (LODs) were in the range of 0.11–0.16 μg mL^−1^, while the limit of quantification (LOQ) values were between 0.35 and 0.51 μg mL^−1^.

**Figure:**
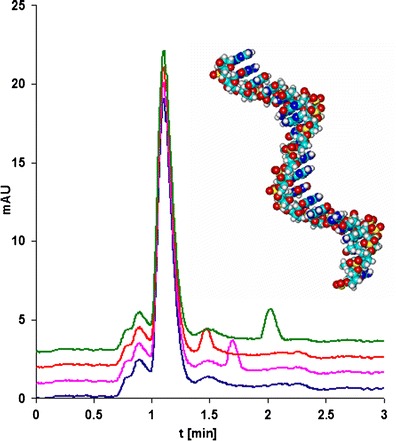
Determination of antisense phosphorothioate oligonucleotides in serum

Determination of antisense phosphorothioate oligonucleotides in serum

## Introduction

Oligonucleotides are components of nucleic acids, which are built of nucleotide monomers [1, 2]. They play an important role in most living organisms. They may be divided into unmodified and modified oligonucleotides [1, 2]. The latter are synthetic compounds with a modification of either the nitrogen base or phosphate group. These alterations may be connected by introduction of the methyl group at each phosphorus in the oligonucleotide chain or at the bases; consequently, methylphosphonates are synthesized [1]. However, phosphorothioates are the most popular [1]. Their structure is modified by the replacement of one of the nonbridging oxygens by sulfur at each phosphorus in the chain. Such modifications improve their enzymatic stability and cell membrane permeability, as phosphorothioate oligonucleotides are used in antisense therapy [1, 2]. On the other hand, utilization of unmodified oligonucleotides is limited. They are applied for the synthesis of deoxyribonucleic and ribonucleic acids or for polymerase chain reactions [1, 2].

Many analytical challenges are involved in analyzing oligonucleotides when their purity, quality, and therapeutic action are considered and studied [3]. The development of analytical methods for sensitive, quantitative analysis of oligonucleotides has been the purpose of many investigations [3]. Methods commonly used for the analysis of oligonucleotides include polyacrylamide gel electrophoresis, capillary electrophoresis [4], ion-pair chromatography [1, 5, 6], and anion-exchange high-performance liquid chromatography [1, 7, 8]. Liquid chromatography enables high-throughput analysis, is relatively inexpensive, easy to use, and precise [1, 5–8]. Therefore, this technique has become more widespread in the analysis of oligonucleotide purity or the fate of antisense oligonucleotides in the organism [1, 5–8]. The difference in retention behavior between oligonucleotides of the same length appears to be based on various hydrophobicity (nitrogen base-dependent) or various charges of all the base pairs in the sequence [5–8]. High-performance liquid chromatography allows to obtain good resolution of the studied compounds; however, the time of analysis may be long. Recently, an ultrahigh-performance liquid chromatography (UHPLC) method was utilized to improve the speed and resolution of oligonucleotide separation [9–11]. It was applied in the analysis of short-interfering RNA. RNA and its metabolites were resolved by UHPLC, while utilization of mass spectrometry allowed for confirmation of their identity [10]. Reversed-phase ion-pairing chromatography was used in these investigations. This method has become a standard separation platform for the analysis of oligonucleotides [5, 6, 9–11]. Due to the negatively charged phosphate groups along the oligonucleotide backbone, they are retained on the stationary phase surface when the ion-pair chromatography (IPC) mode is used [11]. Many different ion-pair reagents (which attract the negatively charged groups) were studied for chromatographic analysis of these compounds [5, 9, 11]. IPC with the use of UHPLC was also used for the study of oligonucleotide therapeutics and their sequence-related impurities. The use of multiple ion-pairing agents in the mobile phase can, in some cases, improve the overall chromatographic resolution and peak symmetry [11]. Consequently, identifying related impurities may be confirmed with greater certainty [11]. Generally, UHPLC was shown to be an effective tool for a fast and efficient analysis of unmodified and modified oligonucleotides [9–11] and for sequence confirmation of complex oligonucleotide mixtures. However, all of the investigations were performed with the use of the conventional, octadecyl stationary phase [5, 9–11]. Utilization of other UHPLC columns can provide greater selectivity.

The main aim of the present investigation was to apply various UHPLC columns for the analysis of unmodified and phosporothioate oligonucleotides. The influence of the type and concentration of ion-pair reagent on the retention of the studied biomolecules was tested. Moreover, different stationary phases were investigated. The developed methods were used to separate unmodified oligonucleotides and to determine antisense oligonucleotides in human serum samples.

## Materials and methods

### Materials

All of the oligonucleotides used in our investigations were purchased from Sigma-Aldrich (Gillingham, Dorset, UK). Table 1 presents the sequences and main physicochemical properties of the studied compounds. Two different groups can be distinguished: unmodified oligonucleotides, phosphodiesters, and modified oligonucleotides (phosphorothioates), in which a sulfur atom was introduced to each phosphate group in the oligonucleotide’s backbone during synthesis. After the synthesis process, the oligonucleotides were purified by RP HPLC and were provided in a lyophilized form. Standard solutions were prepared by dissolution in deionized water at a concentration of 0.1 mM.

**Table 1:** The sequences and molecular masses of oligonucleotides used in the study

Shortcut	Sequence	Molecular weight [g mol^−1^]	*T*_m_ [°C]
OL1	5′-ATCGATCGATCGATCGATCG-3′	6,113	45.4
OL2	5′-ATCGATCGATCGATCGATC**A**-3′	6,098	43.4
OL3	5′-ATCGATCGATCGATCGATC**C**-3′	6,074	45.4
OL4	5′-ATCGATCGATCGATCGA**A**CG-3′	6,122	45.4
OL5	5′-ATCGATCGA**A**CGATCGATCG-3′	6,122	45.4
OL6	5′-ATCGATCGA**G**CGATCGATCG-3′	6,147	47.5
OL7	5′-ATCGATCGAT**A**GATCGATCG-3′	6,137	43.4
OL8	5′-ATCGA**A**CGATCGATCGATCG-3′	6,122	45.4
OL9	5′-ATCGATCGATCGATCGATC**T**-3′	6,147	43.4
AS1	5′-TCTCCCAGCCTGCCCAT-3′	5,687	72.9
AS2	5′-CGGCATGTCTATTTTGTA-3′	6,371	74.9
AS3	5′-TATCCGGAGGGCTCGCCATGCTGCT-3′	8,039	80.2
AS4	5′-GCCCAAGCTGGCATCCGTCA-3′	5,772	54.4
AS5	5′-TCCGTCATCGCTCCTCAGGG-3′	6,353	72.1
AS6	5′-GTTCTCGCTGGTGAGTTTCA-3′	6,438	63.1

Mobile phases were prepared with the use of acetonitrile of gradient-grade purity (J. T. Baker, Deventer, Holland) and deionized water, taken from Milli-Q system (Millipore, El Passo, TX, USA). Moreover, high-purity ion-pair reagents were also used. Triethylammonium acetate buffer (1 M solution), *N*,*N*-dimethylbutylamine, and acetic acid were obtained from Sigma-Aldrich (Dorset, UK).

### Apparatus and analysis conditions

The UltiMate® 3000 Binary Rapid Separation LC (RSLC) (Dionex, Sunnyvale, CA, USA) UHPLC system including a binary pump and a DAD-3000RS Diode Array Detector were used during research. The pump works at high backpressure up to 800 bar. All data were collected with the use of Chromeleon 7.

In the current study, six different commercial UHPLC columns were used. The type of stationary phases, size of silica particles, column dimensions, and manufacturer are presented in Table 2. The operating pressures for the UHPLC columns were in the ranges of: 480–680 bar for Kinetex and 520–730 bar for Hypersil.

**Table 2:** The types of stationary phases used in the investigations together with silica particle size and column dimensions

Abbreviation	Column description	Stationary-phase type	Silica particle size [μm]	Pore diameter [Å]	Column dimensions [mm]	Carbon content [%]	Manufacturer
C1	Kinetex C18 Core-Shell	Octadecyl	1.3	100	50 × 2.1	10	Phenomenex, CA, USA
C2	Kinetex C18 Core-Shell	Octadecyl	1.7		100 × 2.1	12	Phenomenex, CA, USA
C3	Kinetex PFP Core-Shell	Pentafluoro phenyl				9	Phenomenex, CA, USA
C4	Hypersil GOLD C18	Octadecyl	1.9	175		10	Thermo Scientific, CA, USA
C5	Hypersil GOLD C8	Octyl				8	Thermo Scientific, CA, USA
C6	Hypersil GOLD Phenyl	Phenyl				8	Thermo Scientific, CA, USA

Retention studies were carried out in the isocratic (retention studies) or gradient (separation attempts) elution mode. Mixtures of acetonitrile and solutions of ion-pair (IP) reagents were utilized. The acetonitrile content in the mobile phase was in the range of 10–15 % *v*/*v*. Two IP reagents were tested: triethylammonium acetate (TEAA) and *N*,*N*-dimethylbutylamine acetate (DMBAA). TEAA was prepared by diluting 1 M of preformulated commercial buffer. DMBAA was prepared by adding an appropriate amount of amine to water (pH of the solution was in the range of 10 to 12) and adjusting the pH to 6.8–7.0 with glacial acetic acid.

The flow rate was 0.3 mL min^−1^. The void (*t*_0_) of the column was measured by methanol injection. The detection wavelength was selected as *λ* = 260 nm. The injection volume was 0.1 μL (for the retention studies) or 0.5 μL (for separation attempts). The temperature of the autosampler and column was 30 °C.

### Quantification of phosphorothioate oligonucleotides in serum

The phosphorothioate oligonucleotides were quantified on the basis of a calibration curve prepared with six calibration standards of different concentrations. The human serum samples were spiked with standard solutions of the analyzed compounds. Solutions for the calibration curve were prepared by dilution with serum. Each standard solution was analyzed in triplicate. Concentrations of the antisense oligonucleotides were in a range of 0.1 to 21.3 μg mL^−1^. Limits of detection (LODs) and quantification (LOQs) were determined with the use of chromatograms obtained for serum samples fortified with a known amount of phosphorothioate oligonucleotides. The LOD was the lowest concentration providing signal-to-noise ratios (S/N) of about 3, while the LOQ was equal to S/N = 10.

Five different known concentrations of the studied oligonucleotides (range from 0.2 to 21 μg mL^−1^) in the human serum were analyzed in order to calculate the accuracy of the method. Next, accuracy was calculated as the percentage error of theoretical versus measured concentrations. Precision of the method was estimated on the basis of repeatability and intermediate precision (intra-day and inter-day reproducibility). These were determined by triplicate analysis of samples containing phosphorothioate oligonucleotides at concentrations ranging between 0.2 and 20 μg mL^−1^. These concentrations were expressed as the coefficient of variation (CV) of the analyses.

## Results and discussion

### Selection of oligonucleotides

Two groups of oligonucleotides were chosen for the present investigation: unmodified and modified ones (Table 1). The former are typical compounds which can be found in an organism or can be applied for PCR (OL1–OL9 in Table 1). All nine compounds were of the same length and differing in both the type and position of one nucleotide in the sequence. Replacement of one base with another caused difficulty in their separation by conventional HPLC. Since UHPLC has greater resolution power, these oligonucleotides were chosen for testing system efficiency in their separation.

Oligonucleotides with a modification in each phosphate group in the backbone were also studied (AS1–AS6 in Table 1). These were phosphorothioate oligonucleotides which belong to antisense compounds used as therapeutic agents [1, 2, 12]. This group of antisense compounds is the most widely studied because of their nuclease stability and relative ease of synthesis [1, 12]. We selected six different phosphorothioate oligonucleotides which are in the second and third phases of clinical testing [1, 2, 12]. They have the potential to treat leukemia and various other blood cancers (AS1), non-Hodgkin’s lymphoma (AS2), pancreatic cancer (AS3), inflammatory bowel disease, pouchitis, asthma (AS4), glioblastoma (AS5), and corneal graft rejection (AS6) (Table 1).

From an analytical point of view, the selection of unmodified and antisense oligonucleotides allows to determine the influence of modification in the structure on retention in ion-pair chromatography. Moreover, the ability of determining phosphorothioate oligonucleotides using UHPLC will also be investigated.

### Type of stationary and mobile phases

UHPLC is becoming a commonly used technique for the separation and determination of biologically active compounds due to the resolution power this system provides. This effect is a result of the utilization of stationary phases with reduced particle size. In most cases, only one type of packing material is used, namely octadecyl stationary phase. The same concerns utilization of UHPLC in the analysis of oligonucleotides [5, 11]. However, there are at least over a dozen various UHPLC columns with different packing materials, such as octyl, phenyl, or amide. Each one of these may provide greater selectivity than the commonly used octadecyl stationary phase. Therefore, the main aim of the present investigation was to test the effect of stationary phase ligands on the retention and separation of oligonucleotides. Octyl, phenyl, and pentafluorophenyl columns were used for this purpose (Table 2).

Ion-pair chromatography has become a standard separation method for analysis of oligonucleotides due to the negatively charged phosphate or phosphorothioate in their structure [3, 5–7, 10, 11]. Cationic IP reagent is used for oligonucleotide analysis. Triethylamine is most commonly used in the solution of triethylammonium acetate; therefore, we also tested this salt. Moreover, *N*,*N*-dimethylbutylamine acetate was also applied. Both IP reagents differ only in structure because their molecular formula is similar (they are built of six carbon atoms and one nitrogen). The goal of the investigation was to compare how the IP reagent’s structure affected the retention and separation of the tested oligonucleotides.

### Impact of IP reagent concentration on the retention of oligonucleotides

**Fig. 1:**
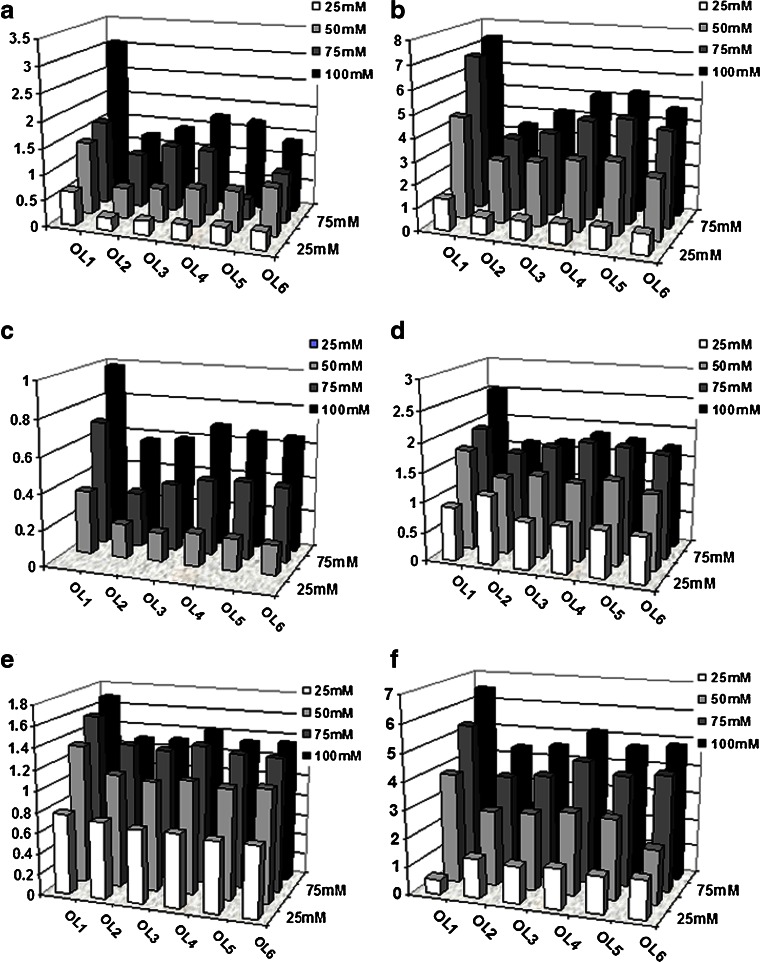
Influence of triethylammonium acetate (TEAA) concentration on the retention of unmodified oligonucleotides for all columns used in the investigations: **a** C1, **b** C2, **c** C3, **d** C4, **e** C5, and **f** C6. Experimental conditions: mobile-phase composition: 10 % *v*/*v* ACN, 90 % *v*/*v* TEAA; flow rate, 0.3 mL min^−1^; autosampler and column temperature, 30 °C; UV-Vis detection, 260 nm; and injection volume, 0.1 μL. For notation of oligonucleotides, see Table 1

The first step of the investigations involved studying the influence of the IP reagent concentration on oligonucleotide retention. The concentration of the IP agent is critical to the amount of retention experienced by the oligonucleotide. Figure 1 presents the dependencies obtained for all columns and six unmodified oligonucleotides. These results were determined for mobile phases containing TEAA. As can be observed, the retention factor (*k*) values have greater values when the concentration of TEAA increases. This phenomenon confirms the occurrence of an ion-pairing mechanism and reflects the increase in efficiency of ion-pair formation during the chromatographic process. Consequently, retention of polar oligonucleotides increases as a result of mix-mode interactions between alkylamine (TEA^+^) and both stationary phase and the studied biomolecules.

Oligonucleotides were retained on the stationary phase surface to the greatest extent in columns C2 and C6 (Fig. 1b, f). Octadecyl and phenyl stationary phases were packed into these two columns, respectively. Surprisingly, other octadecyl columns used in the investigations (C1 and C4) allowed to obtain lower *k* values in comparison with C2 (Fig. 1a, b, d). The main reason for this effect is probably the percentage part of the carbon on the silica support (Table 2). The carbon load is equal to 10 % for columns C1 and C4 (Table 1); therefore, the retention of oligonucleotides for both columns is similar (Fig. 1a, d). Column C2 possesses a higher carbon content at the surface of the stationary phase; hence, ion pairing is more effective for this packing material (Table 1). The *k* values for the octyl stationary phase are lower than those for the octadecyl packings (Fig. 1e). However, the alkyl chain on the support surface is shorter; thus, hydrophobicity and carbon load are also below values determined for octadecyl columns (Table 2). Utilization of the pentafluorophenyl packing material provided the lowest *k* values among all of the studied columns (Fig. 1c). This type of stationary phase possesses a different selectivity as compared to the alkyl phases through other interactions (π-π interactions, dipole-dipole, and hydrogen bond interactions) affecting the chromatographic behavior of the analytes. Various chromatographic performances are commonly attributed to the polarity of the CF bond. Although retention of oligonucleotides was low for C3, differences in the *k* values for the various biomolecules analyzed with the same concentration of TEAA are noticeable and may allow to use C3 for the separation of studied oligonucleotides (Fig. 1c).

The C6 column has a similar carbon load as C5 or even lower than that of the rest of the studied columns (Table 2). Despite this phenomenon, the C6 column retains oligonucleotides on the surface of the stationary phase to a great extent (Fig. 1f). The high *k* values of oligonucleotides measured for the phenyl packing material are connected mainly with the ability of π-π interaction between modified support and the analyzed biomolecules (Fig. 1f). Such interactions also appear in the DNA and RNA structures and are responsible for the so-called base-stacking.

The elution order is similar for all of the studied octadecyl columns as well as for the phenyl column (Fig. 1a–d, f). The *k* values were highest for OL1; however, OL4 and OL5 were also retained at the stationary phase surface to a great extent (Fig. 1a–d, f). Thymine in positions 10 and 18 of the oligonucleotide sequence was replaced with adenine, and consequently, retention decreased in comparison with OL1 (Table 1). This effect may be surprising because the typical elution order of the nucleotides being analyzed with the use of octadecyl packing material is cytidine < uridine < guanidine < thymidine < adenine. Therefore, substituting thymidine with adenine should increase the *k* values; however, the secondary structure should also be considered and taken into account. OL1 possesses a sequence which favors the creation of stem-loop, intramolecular base pairing (Table 1). The two fragments of the same strand form base pairs of the double helix, which ends in an unpaired loop. Introducing another, different nucleotide (such as adenine) causes a change in the secondary structure and a reduction of *k* values (Fig. 1a–d, f). OL2 and OL3 had the lowest retention in columns C1, C2, C3, and C4. This effect is not connected with the change in the secondary structure of the oligonucleotides since guanidine at the 3′ end of a sequence does not take part in base pairing (Table 1). Replacement of the pyrimidine (G) base with a purine base (A or C) causes an increase in the hydrophilicity of OL2 and OL3, and consequently, they are retained with lower strength by the stationary phase (Fig. 1a–d, f).

The elution order for C5 is similar for all tested oligonucleotides, independently of concentration (Fig. 1e). Utilization of the octyl stationary phase is not suitable for separation of oligonucleotides.

### A comparison of TEA and DMBA ion-pair reagents

**Fig. 2:**
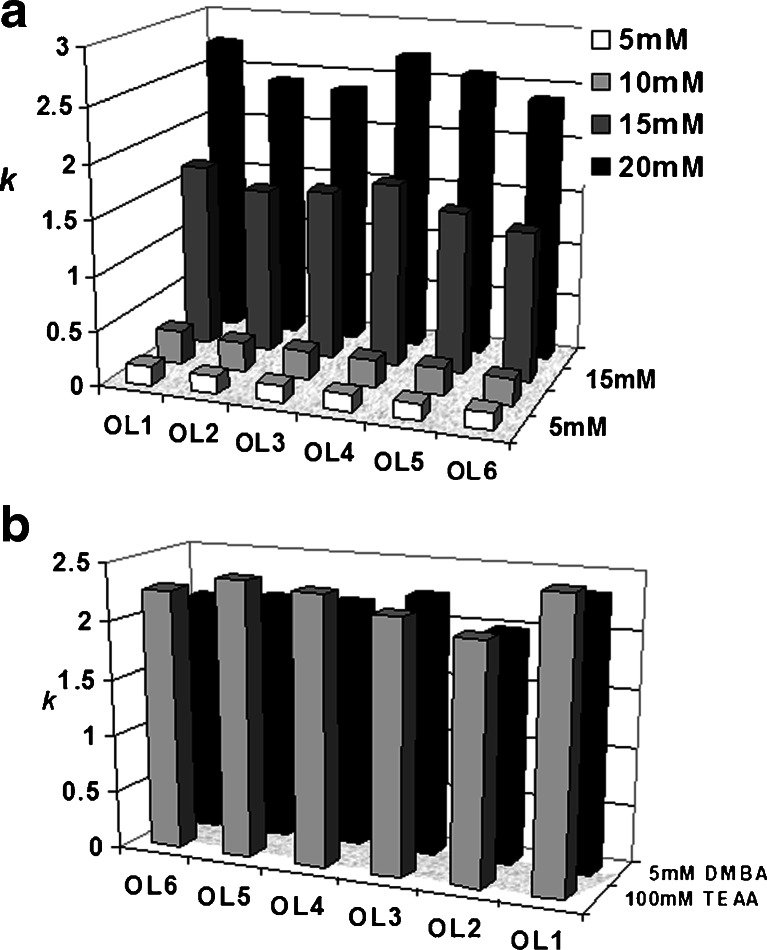
Impact of *N*,*N*-dimethylbutylammonium acetate (*DMBA*) on the retention of oligonucleotides on column C2: **a** influence of the DMBAA concentration on *k* values (mobile-phase composition: 15 % *v*/*v* of acetonitrile, 85 % *v*/*v* of DMBAA), **b** comparison of *k* values determined for 5 mM of DMBA and 100 mM TEAA (mobile-phase composition: 10 % *v*/*v* acetonitrile, 90 % *v*/*v* of IP reagent). Experimental conditions: flow rate, 0.3 mL min^−1^; autosampler and column temperature, 30 °C; UV-Vis detection, 260 nm; and injection volume, 0.1 μL. For notation of oligonucleotides, see Table 1

Dimethylbutylammonium acetate (DMBAA) was also used for retention studies of oligonucleotides in IPC chromatography for all of the columns. Figure 2a presents exemplary results obtained for column C2. Two general trends may be observed: first, the *k* values became higher with an increase in concentration of the IP reagent, and second, the retention of oligonucleotides was greater when DMBAA was used compared to data determined for TEAA (Fig. 1b). The higher content of acetonitrile (15 % *v*/*v*) in the mobile phase was indispensable for the retention studies of oligonucleotides due to the high retention times when DMBAA was used. Similar tendencies were noticed for all of the other columns; therefore, these data will not be included in the manuscript. Figure 2b presents a comparison of *k* for six unmodified oligonucleotides determined in the same percentage part of acetonitrile (10 % *v*/*v*) and in various concentrations of DMBAA and TEAA. It may be observed that *k* values for the oligonucleotides studied here are similar when a 20-fold lower concentration of DMBAA (5 mM) was used as compared to the concentration of TEAA (100 mM). Although DMBA and TEA cations are built of the same carbon atoms, their impact on the retention of the studied biomolecules is different due to the structure of the IP reagents. DMBA possesses a butyl chain in the structure; thus, it can be more effectively bonded to the nonpolar stationary phase through hydrophobic interactions. Oligonucleotides are attracted by the dynamically generated charge on the surface of the support. As a result, the *k* values of the polyanionic compounds studied here are higher in comparison with the use of TEA as an IP reagent.

### The separation of unmodified oligonucleotides

Separation of homologous oligonucleotides built of the same nucleotides and differing only in chain length is the most common attempt when separation techniques are used for analysis of these types of biomolecules. However, it is much more challenging to separate oligonucleotides differing in the position of a single nucleotide or in the modification of the nitrogen base. HPLC rarely gives good results and high resolution for these types of mixtures. Therefore, UHPLC was selected to test its ability to separate mixtures of oligonucleotides built of the same number of mers. Several attempts to separate all nine oligonucleotides were done during the investigations; however, a complete separation of studied biomolecules was not possible, even if the time of analysis was equal 30 min. Three test mixtures were composed of molecules differing in (1) position of one, the same nucleotide (A) in the sequence, (2) position of three nucleotides (A, C, G) in the sequence, and (3) type of nucleotide (A, C, G) in the same position of the sequence (20th).

**Fig. 3:**
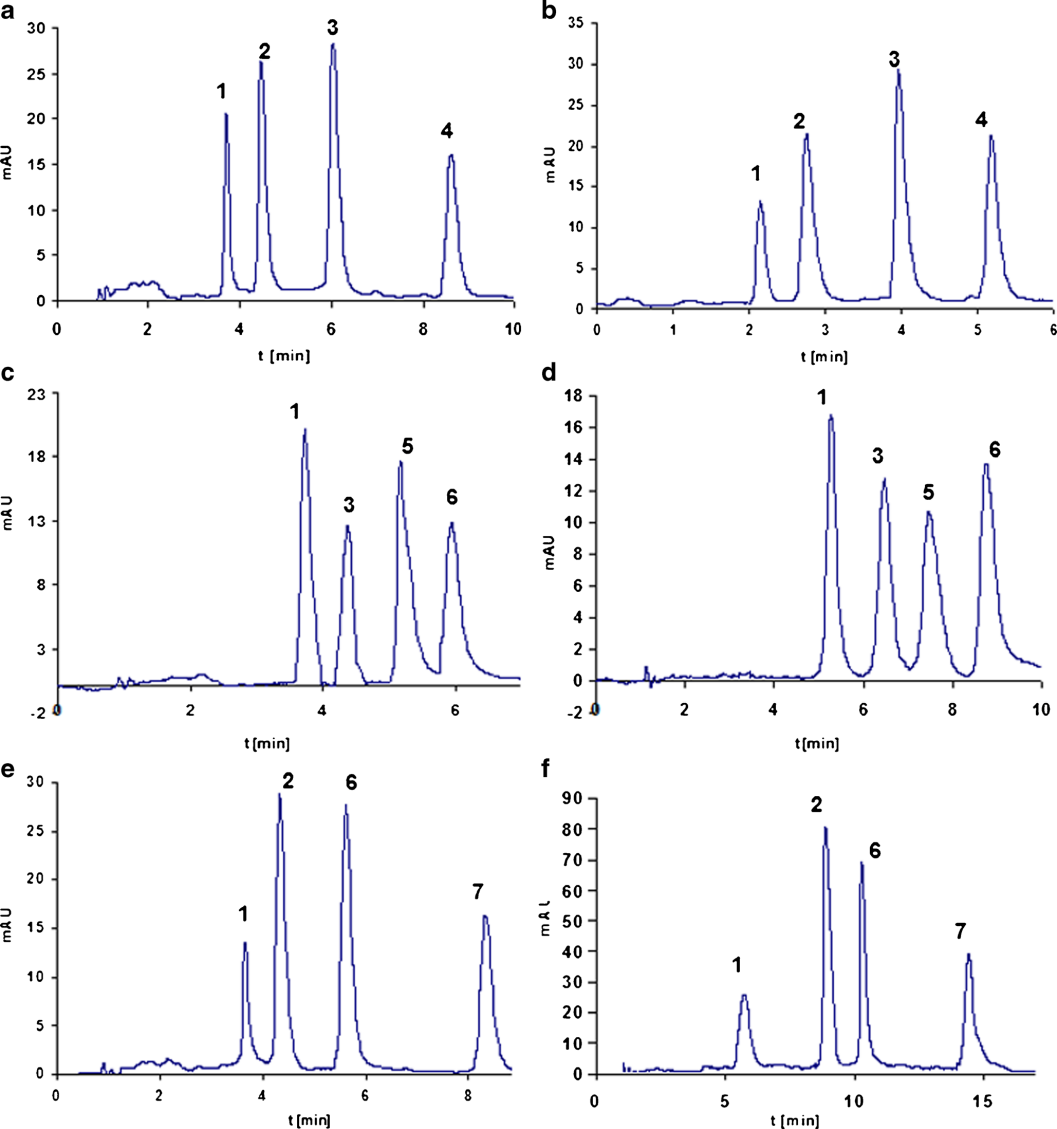
Results of separation of oligonucleotides with the use of various UHPLC columns: **a** C2 (90 % *v*/*v* 100 mM TEAA, 10 % *v*/*v* ACN), **b** C3 (75 mM TEAA, ACN; gradient elution 8–11 % *v*/*v* ACN in 15 min), **c** C2 (91 % *v*/*v* 100 mM TEAA, 9 % *v*/*v* ACN), **d** C6 (91 % *v*/*v* 100 mM TEAA, 9 % *v*/*v* ACN), **e** C2 (90 % *v*/*v* 100 mM TEAA, 10 % *v*/*v* ACN), and **f** C4 (75 mM TEAA/ACN, gradient elution: 8–11 % *v*/*v* ACN in 15 min). Experimental conditions: flow rate, 0.3 mL min^−1^; autosampler and column temperature, 30 °C; UV-Vis detection, 260 nm; injection volume, 0.1 μL. Notation: *1*, OL1, *2*, OL2, *3*, OL3, *4*, OL9, *5*, OL6, *6*, OL8, *7*, OL7. For shortcuts of oligonucleotides, see Table 1

Although the DMBA IP reagent allows for improving retention of oligonucleotides for all of the studied columns, its utilization failed in the separation attempts. Despite the use of isocratic and gradient elution, only three component mixtures of the analyzed biomolecules were separated. The utilization of DMBAA did not provide such a selectivity of resolution as TEAA (Fig. 3).

The best results of separation were achieved when TEAA was used as IP reagent; however, this required a high concentration of this salt (between 75 and 100 mM). Figure 3 presents the results obtained for all of the tested mixtures of unmodified oligonucleotides. The application of octadecyl- and phenyl-based stationary phases allowed for complete resolution of the studied biomolecules in less than 10 min. All of the four columns can be used successfully for the separation of unmodified oligonucleotides. Hydrophobic interactions are the most important and influential during the resolution of polyanionic biomolecules in IPC. On the other hand, π… π interactions also have a great impact on the final result of separation since these types of interactions take place during chromatographic analysis of oligonucleotides on phenyl packings. They also take part in the base-stacking effect that is responsible for the structure of DNA.

The separation of four compound mixtures in 10 min by UHPLC may not seem to be impressive; however, the composition of the sample should be kept in mind, i.e. modifications in the structure of large oligonucleotide molecules are small. Moreover, utilization of HPLC for separation of these types of mixtures would take at least 40 min with gradient elution; therefore, using the UHPLC technique allows to obtain better results.

### UHPLC analysis of antisense oligonucleotides

UHPLC appears to be a very effective analytical tool for the analysis of unmodified oligonucleotides. However, phosphorothioate oligonucleotides are under intensive investigation due to their high solubility and excellent antisense activity. Both quantitative and qualitative analyses are of great importance when studying these compounds.

Based on the results obtained for unmodified oligonucleotides, four UHPLC columns were chosen for their utilization in the analysis of antisense phosphorothioate oligonucleotides. Two phenyl columns, C3 and C6, and two octadecyl columns, C2 and C4, were selected. It has to be pointed out that the selection was done based on retention properties and selectivity, because *k* values were studied for each of column. Moreover, selectivity was also investigated during separation attempts.

**Fig. 4:**
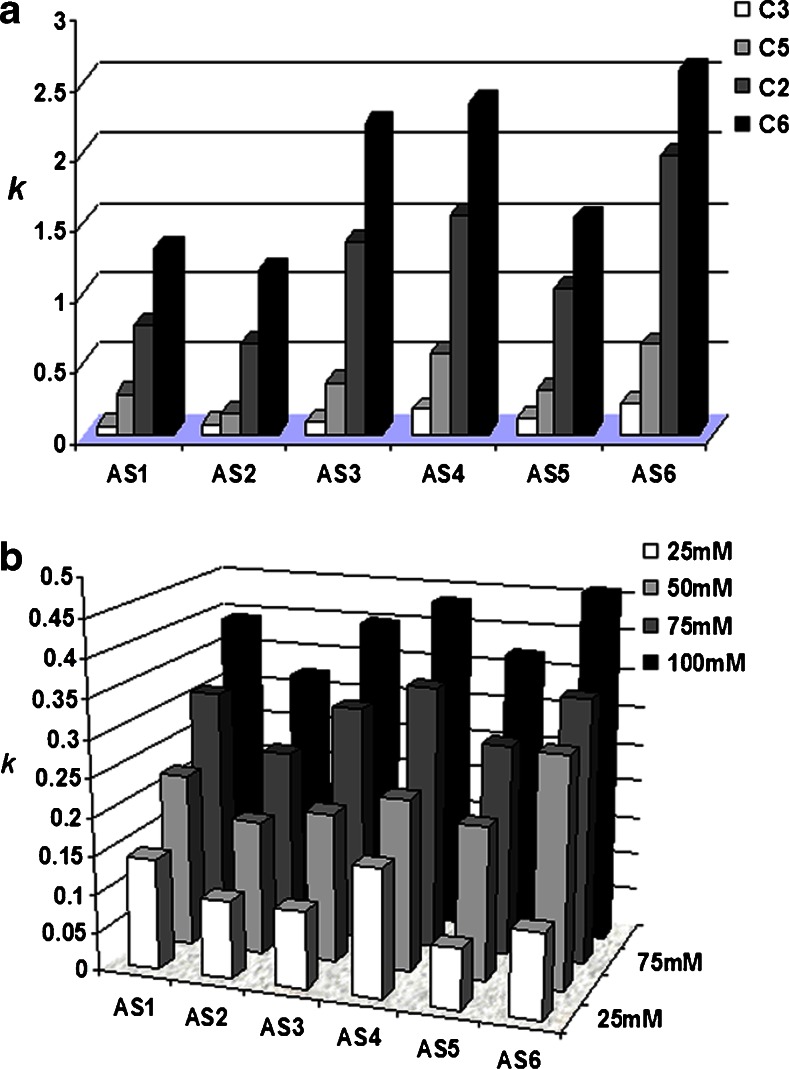
Results of chromatographic analysis of phosphorothioate oligonucleotides with various UHPLC columns: **a** changes of *k* values for antisense oligonucleotides analyzed with the use of four columns (mobile-phase composition: 17 % *v*/*v* of ACN and 83 % *v*/*v* of 100 mM TEAA), **b** impact of TEAA concentration on the retention of antisense oligonucleotides on column C6 (mobile-phase composition: 20 % *v*/*v* of ACN and 80 % *v*/*v* of TEAA). Experimental conditions: flow rate, 0.3 mL min^−1^; autosampler and column temperature, 30 °C; UV-Vis detection, 260 nm; injection volume, 0.1 μL. For shortcuts of oligonucleotides and columns, see Tables 1 and 2

Figure 4a presents the results of a comparison of *k* values for antisense oligonucleotides and columns. The lowest retention was determined for the pentafluorophenyl stationary phase, while the greatest for the phenyl phase (Fig. 4a). The *k* values for the octadecyl packing material were in the range of 1.0–1.9. Moreover, retention determined for 18 mer AS1 and AS2 was low (Fig. 4a). This is connected with the short length of the oligonucleotides and with secondary structure effects. AS1 and AS2 do not form stable hairpin loops; furthermore, if the loop is created, it may be formed only by one base pair. Consequently, this effect does not influence the retention of oligonucleotides. For compounds with a longer phosphorothioate backbone (AS3, AS4, AS5, AS6), *k* values are even two times higher than those for AS1 and AS2 (Fig. 4a). Surprisingly, retention is greater for AS4 and AS6 (20 mer) than that for AS3 (25 mer) (Table 1, Fig. 4a). This effect is connected, again, with the secondary structure of the biomolecules studied in this paper. All of them may form hairpin loops in analysis conditions; therefore, a complete explanation of the differences in their retention factor values is difficult.

On the basis of the results presented in Fig. 4a, it may be concluded that octadecyl C2 and phenyl C6 may be successfully used for the analysis of antisense oligonucleotides. In the next step of investigations, C6 was selected and used to determine the impact of the IP reagent on the *k* values of phosphorothioate oligonucleotides. Figure 4b presents these results. Increasing the IP reagent concentration caused an increase in *k* values. Moreover, 75 or 100 mM of TEAA should be used in routine chromatographic analysis of oligonucleotides since the peak symmetry was high (*f*_AS_ in the range of 0.9–1.1).

### Quantification of antisense phosphorothioate oligonucleotides in serum

**Fig. 5:**
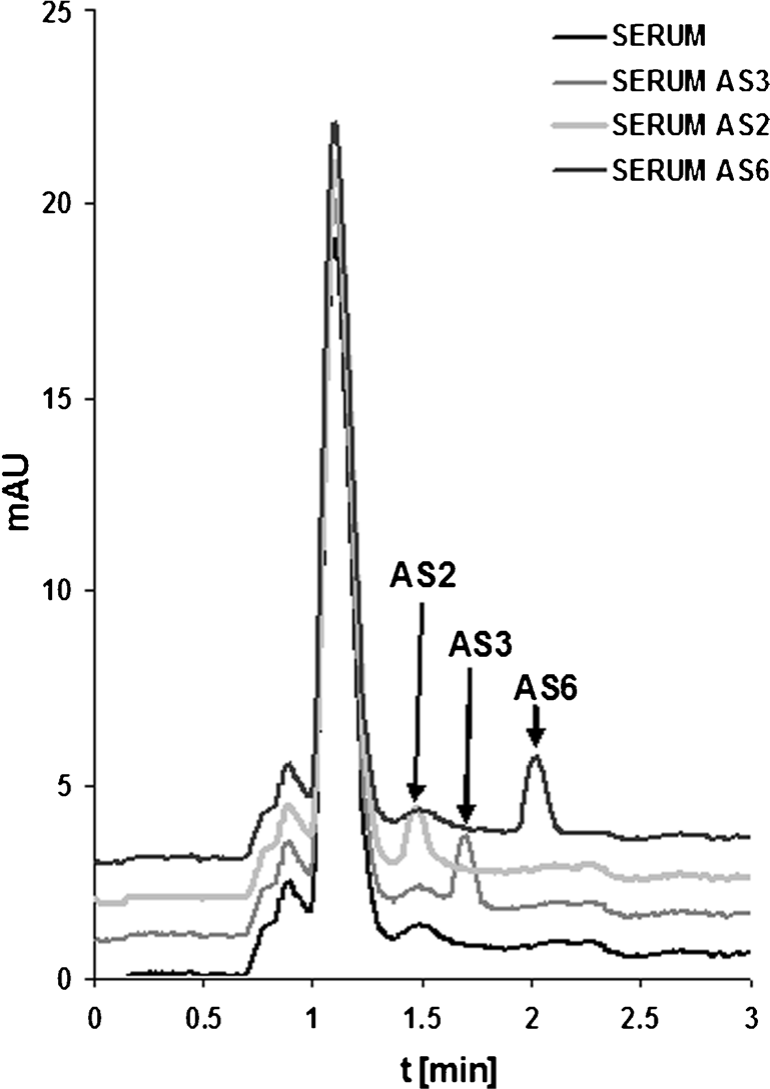
Exemplary chromatograms of serum sample fortified with three phosphorothioate oligonucleotides. Chromatographic conditions: column C6; mobile-phase composition, 20/80 *v*/*v* ACN/100 mM TEAA; flow rate, 0.3 mL min^−1^; injection volume, 0.3 μL; autosampler and column temperature, 30 °C; UV-Vis detection, 260 nm

Phosphorothioate oligonucleotides are currently under clinical tests regarding their antisense activity. Development of a method for their quantification in biological matrices is of great importance; therefore, serum was selected as a biological fluid in which the studied compounds were determined because they will be quantified in such samples when antisense oligonucleotides are used in therapy. Figure 5 presents exemplary chromatograms for three selected oligonucleotides determined in spiked serum with the use of column C6 (Table 2).

Validation of the method was performed with regard to selected parameters, such as linearity, LOD, LOQ, accuracy, and precision. All of the calculated parameters are presented in Table 3. Linearity was determined by calculating the correlation coefficient (*R* ^2^) and slope for the calibration curves. The calibration plots showed good linearity (*R* ^2^ higher than 0.998) within the test ranges (Table 3). Intra-day CV of the calibration curve slopes was in the range of 1.6 to 4.2 %. The LODs were in the range of 0.11–0.16 μg mL^−1^, while the LOQ values were between 0.35 and 0.51 μg mL^−1^ (Table 3). Low concentrations of phosphorothioate oligonucleotides may be successfully determined in serum samples. The inter-day CV ranged from 0.5 to 3.9 %. Accuracy varied with concentration, but was normally in the range of 1.3 to 5.2 %. We have shown that quantification of antisense oligonucleotides in serum was of good accuracy and precision. The results presented in this paper demonstrate the practical ability of UHPLC with DAD detection in determining the biomolecules studied here during a very short period of time (less than 3 min).

**Table 3:** The linearity (expressed as determination coefficient *R* ^2^), slope, and limits of detection and quantification for the determination of phosphorothioate oligonucleotides in serum

Oligonucleotide	Concentration range [μg mL^−1^]	*R* ^2^	Slope	LOD [μg mL^−1^]	LOQ [μg mL^−1^]
AS1	0.15–15.41	0.998	0.2633 ± 0.0131	0.15	0.51
AS2	0.11–16.51	1.000	0.3154 ± 0.0121	0.11	0.38
AS3	0.15–18.24	0.998	0.2799 ± 0.0108	0.15	0.52
AS4	0.16–15.33	0.999	0.4583 ± 0.0151	0.16	0.52
AS5	0.16–16.21	0.999	0.2984 ± 0.0092	0.16	0.51
AS6	0.12–14.68	0.998	0.3754 ± 0.0112	0.12	0.41

## Conclusions

It was demonstrated that UHPLC is well suited for the separation of unmodified oligonucleotides, which is extremely important for purity determination or for monitoring the efficiency of chemical synthesis. The octadecyl and phenyl UHPLC columns are the most suitable for the separation of oligonucleotides differing in the position of one base in the sequence in a time period that is less than 10 min. This is very difficult to achieve with the use of HPLC. Moreover, TEAA should be used instead of DMBAA for the analysis of oligonucleotides when their complete resolution is important. Advanced UHPLC column efficiency provided symmetrical peaks of both unmodified and modified oligonucleotides. The utilization of phenyl UHPLC columns allowed to determine phosphorothioate antisense oligonucleotides in less than 3 min in human serum samples. Furthermore, linearity, LOD, and LOQ had satisfactory values.

## Abbreviations

AS: Antisense oligonucleotide
DMBAA: *N*,*N*-dimethylbutylamine acetate
IP: Ion pair
LOD: Limit of detection
LOQ: Limit of quantification
OL: Oligonucleotide
TEAA: Triethylammonium acetate
UHPLC: Ultrahigh-performance liquid chromatography
